# Supplementary material for: Dysbiosis of gut microbiota in C57BL/6-Lepem1hwl/Korl mice during microplastics-caused hepatic metabolism disruption
Source: PLoS One. 2025 Nov 20;20(11):e0336627. doi: 10.1371/journal.pone.0336627 (PMC12633882; doi:10.1371/journal.pone.0336627)
Supplement: S1 File — (ZIP) [file pone.0336627.s001.zip › Supporting Information/Original Images_v1.pptx]

## Slide 1
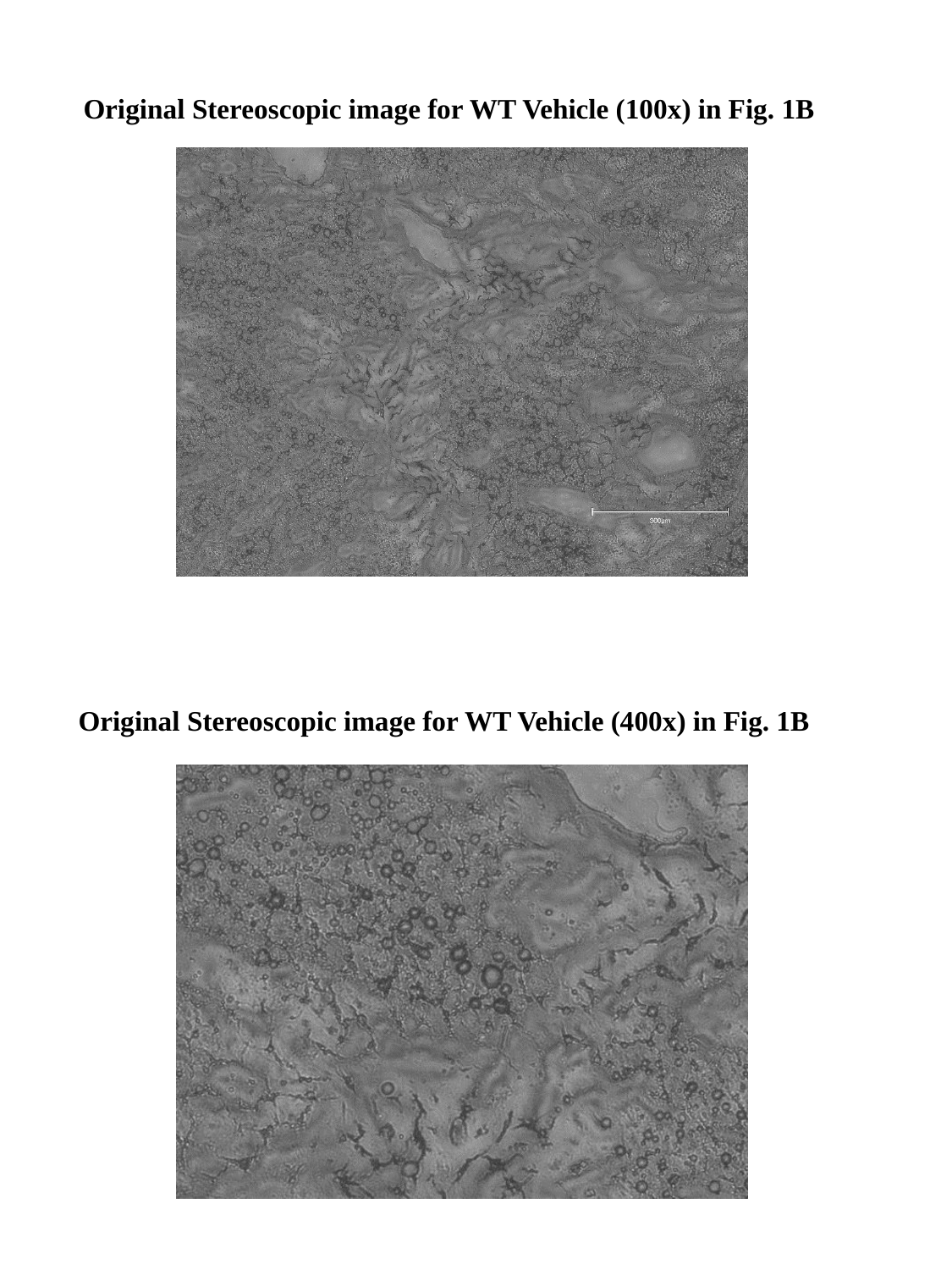

Original Stereoscopic image for WT Vehicle (100x) in Fig. 1B
Original Stereoscopic image for WT Vehicle (400x) in Fig. 1B

## Slide 2
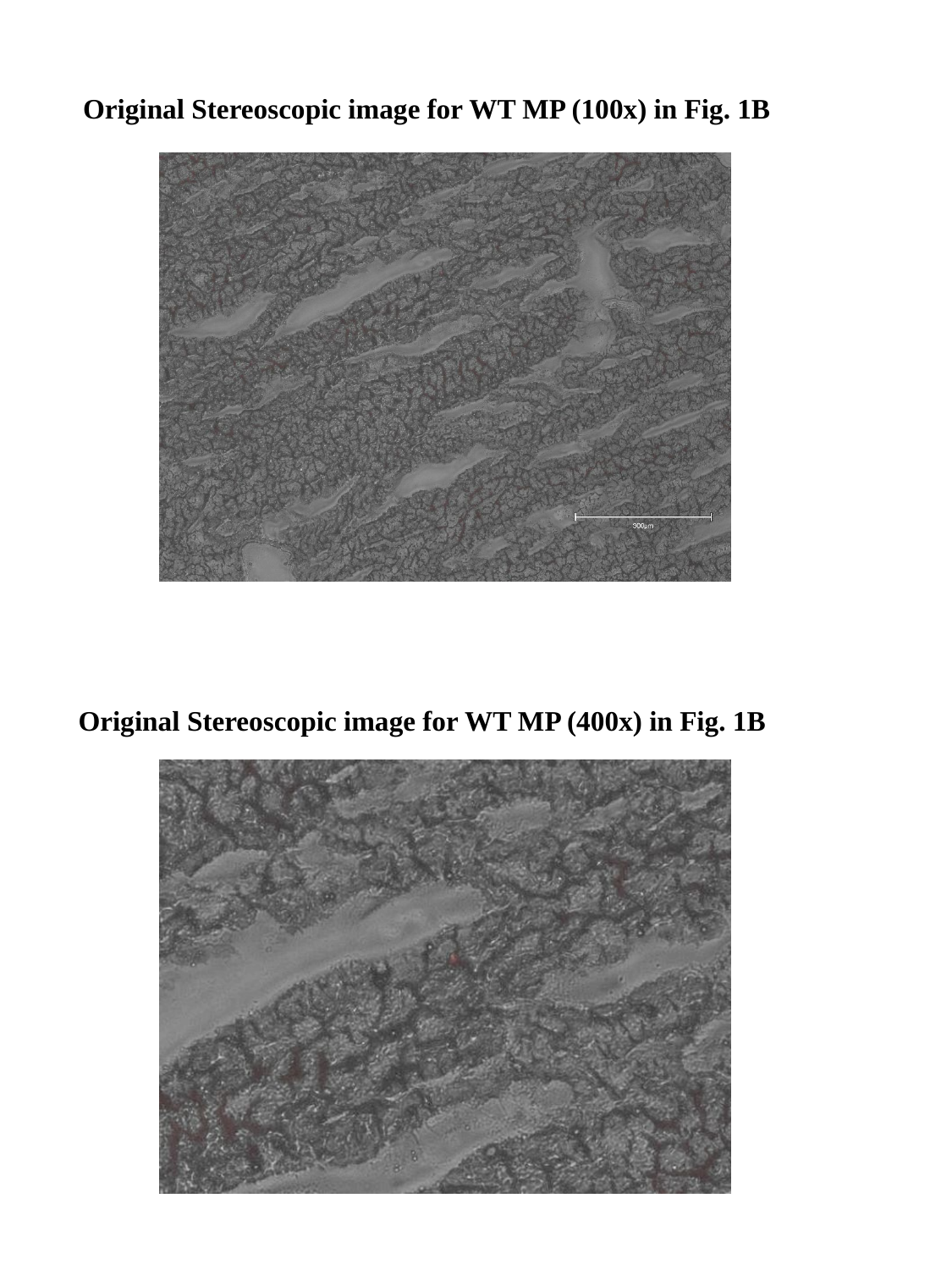

Original Stereoscopic image for WT MP (100x) in Fig. 1B
Original Stereoscopic image for WT MP (400x) in Fig. 1B

## Slide 3
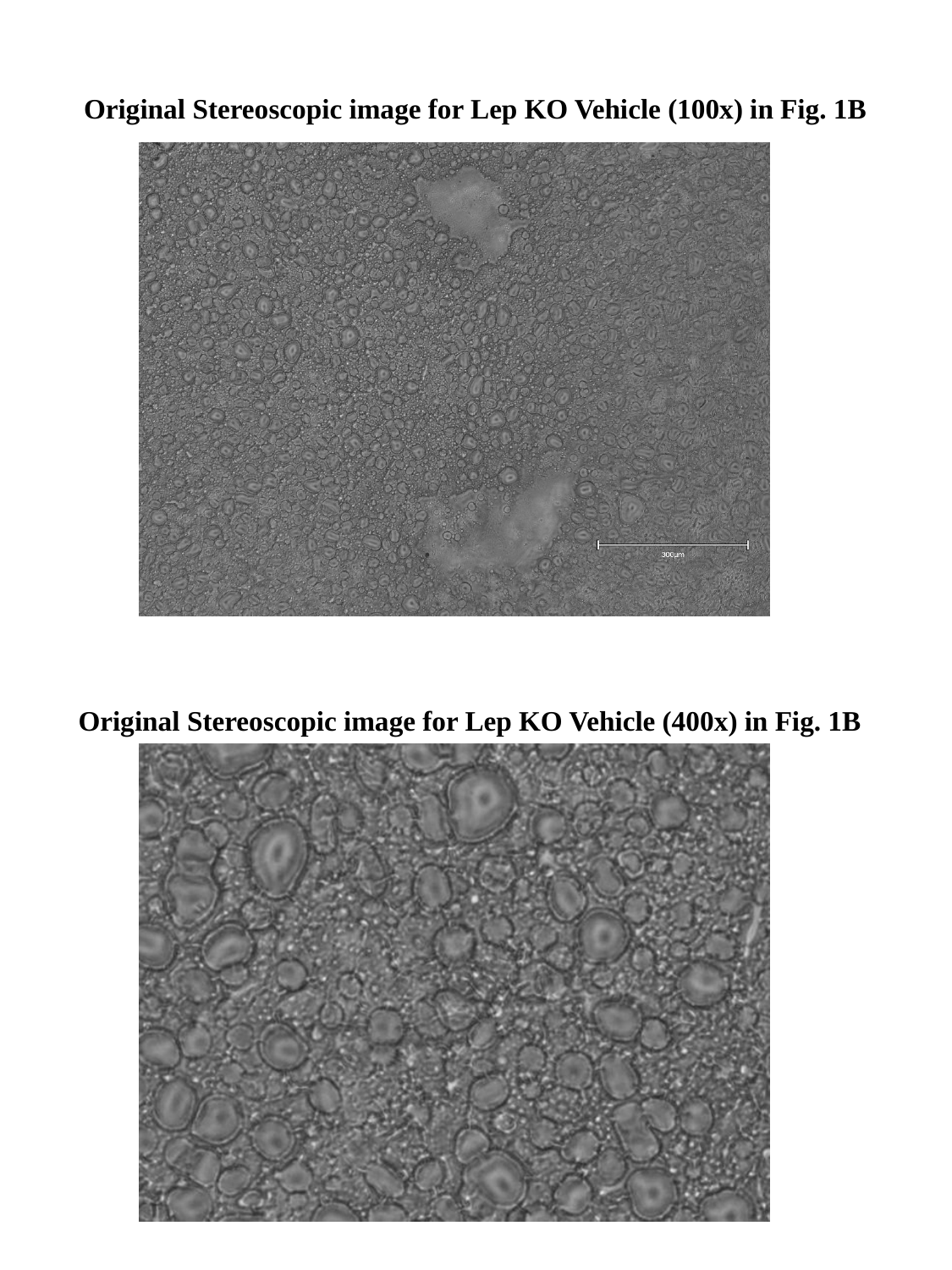

Original Stereoscopic image for Lep KO Vehicle (100x) in Fig. 1B
Original Stereoscopic image for Lep KO Vehicle (400x) in Fig. 1B

## Slide 4
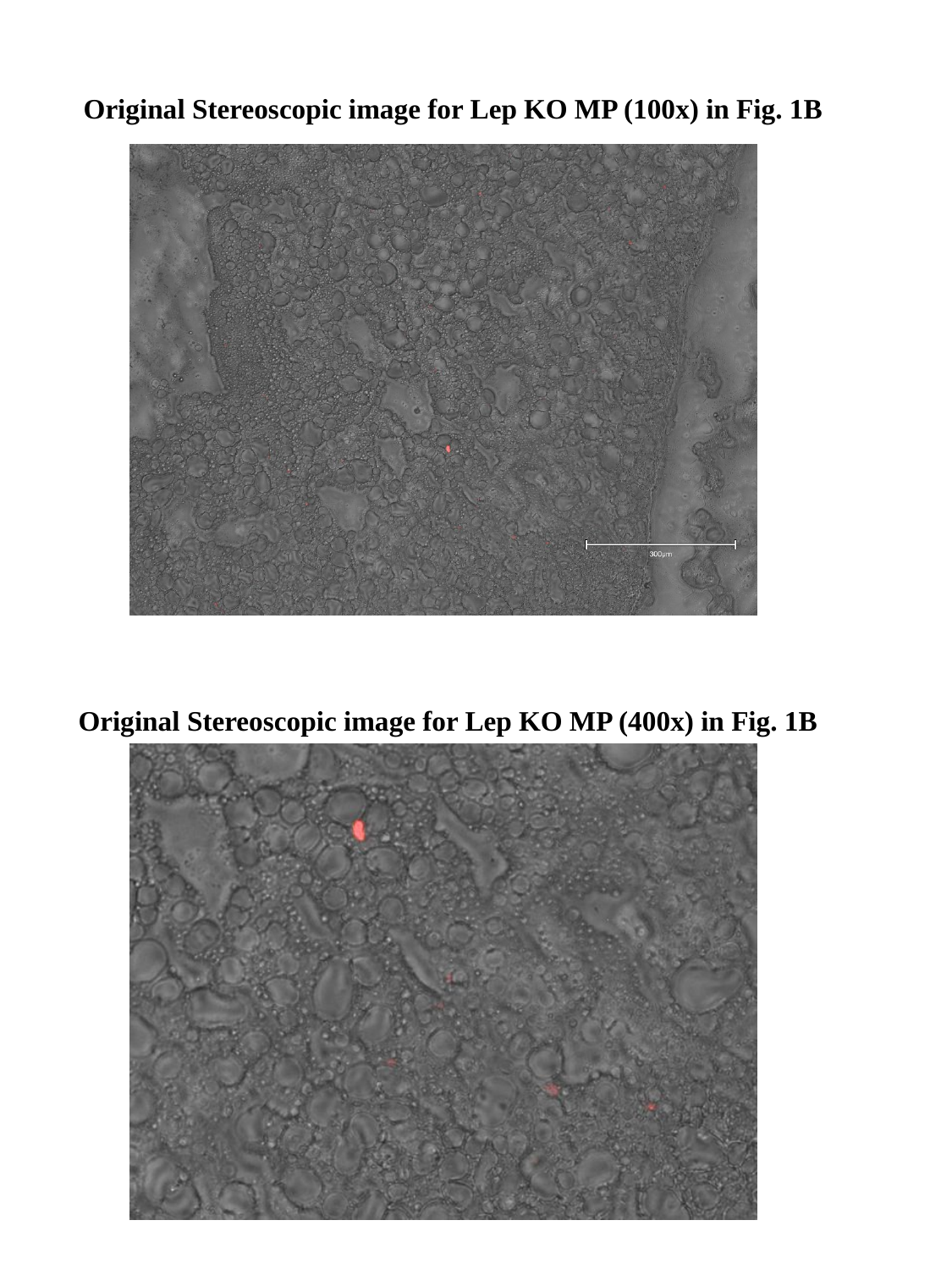

Original Stereoscopic image for Lep KO MP (100x) in Fig. 1B
Original Stereoscopic image for Lep KO MP (400x) in Fig. 1B

## Slide 5
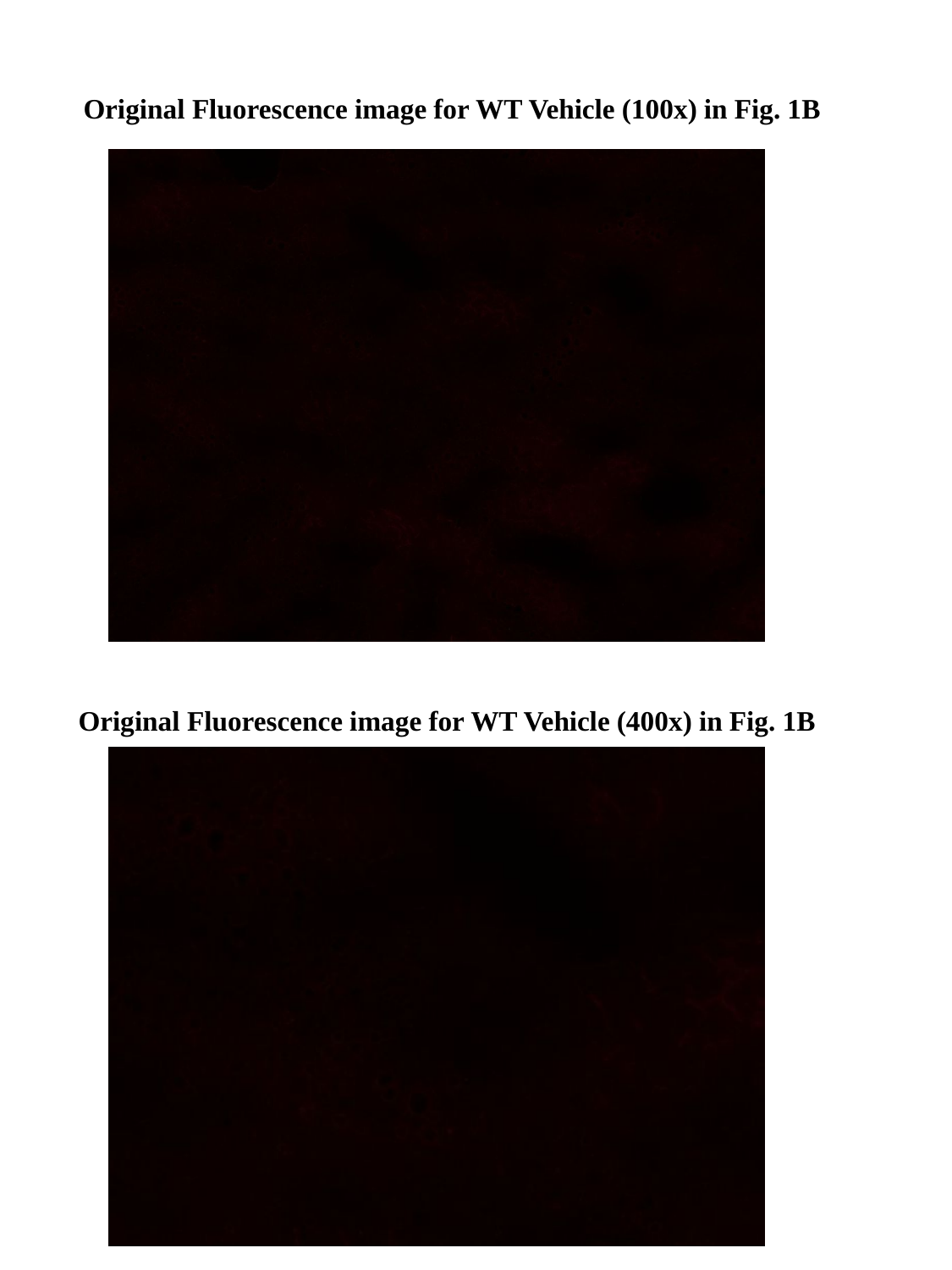

Original Fluorescence image for WT Vehicle (100x) in Fig. 1B
Original Fluorescence image for WT Vehicle (400x) in Fig. 1B

## Slide 6
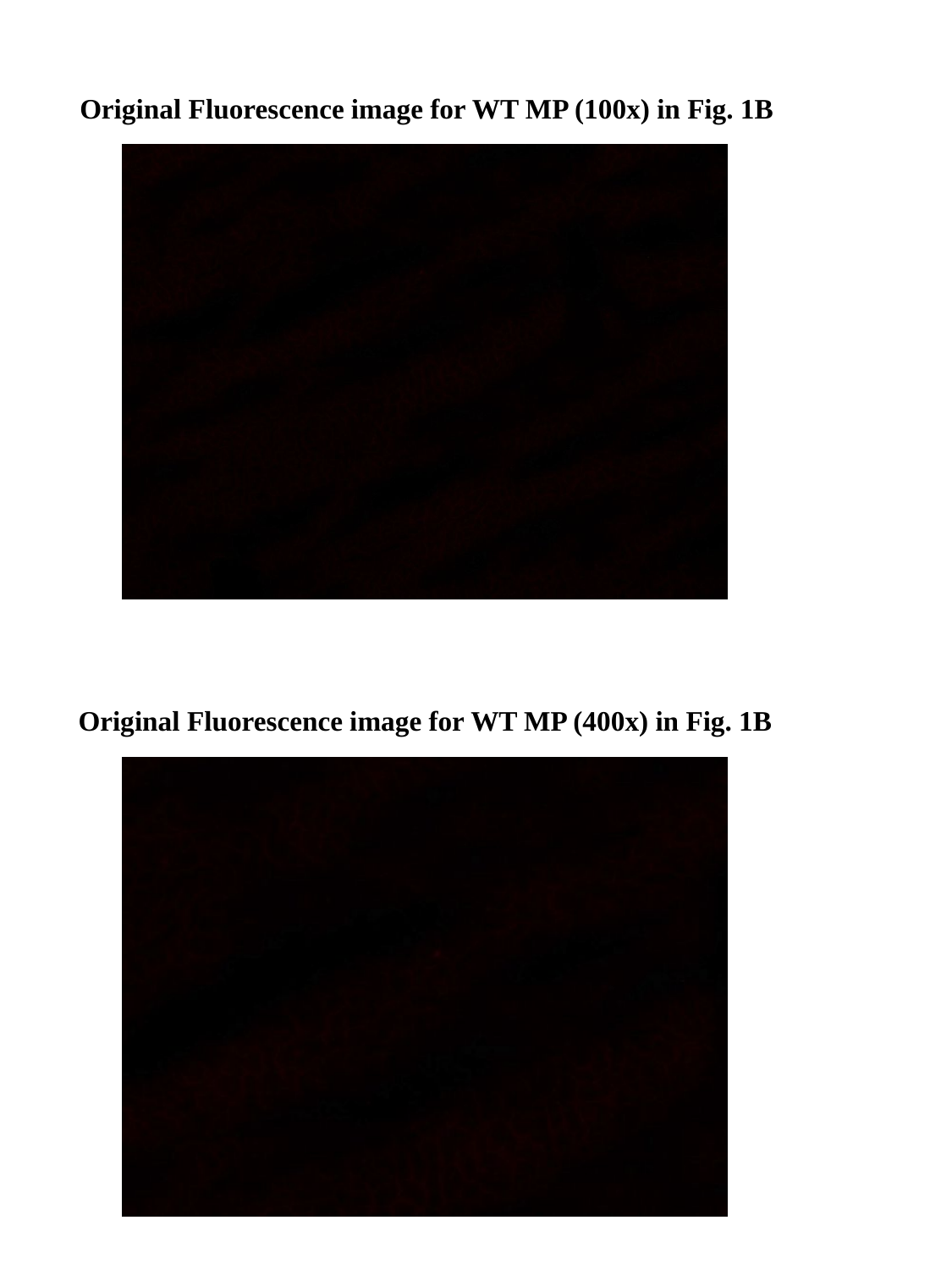

Original Fluorescence image for WT MP (100x) in Fig. 1B
Original Fluorescence image for WT MP (400x) in Fig. 1B

## Slide 7
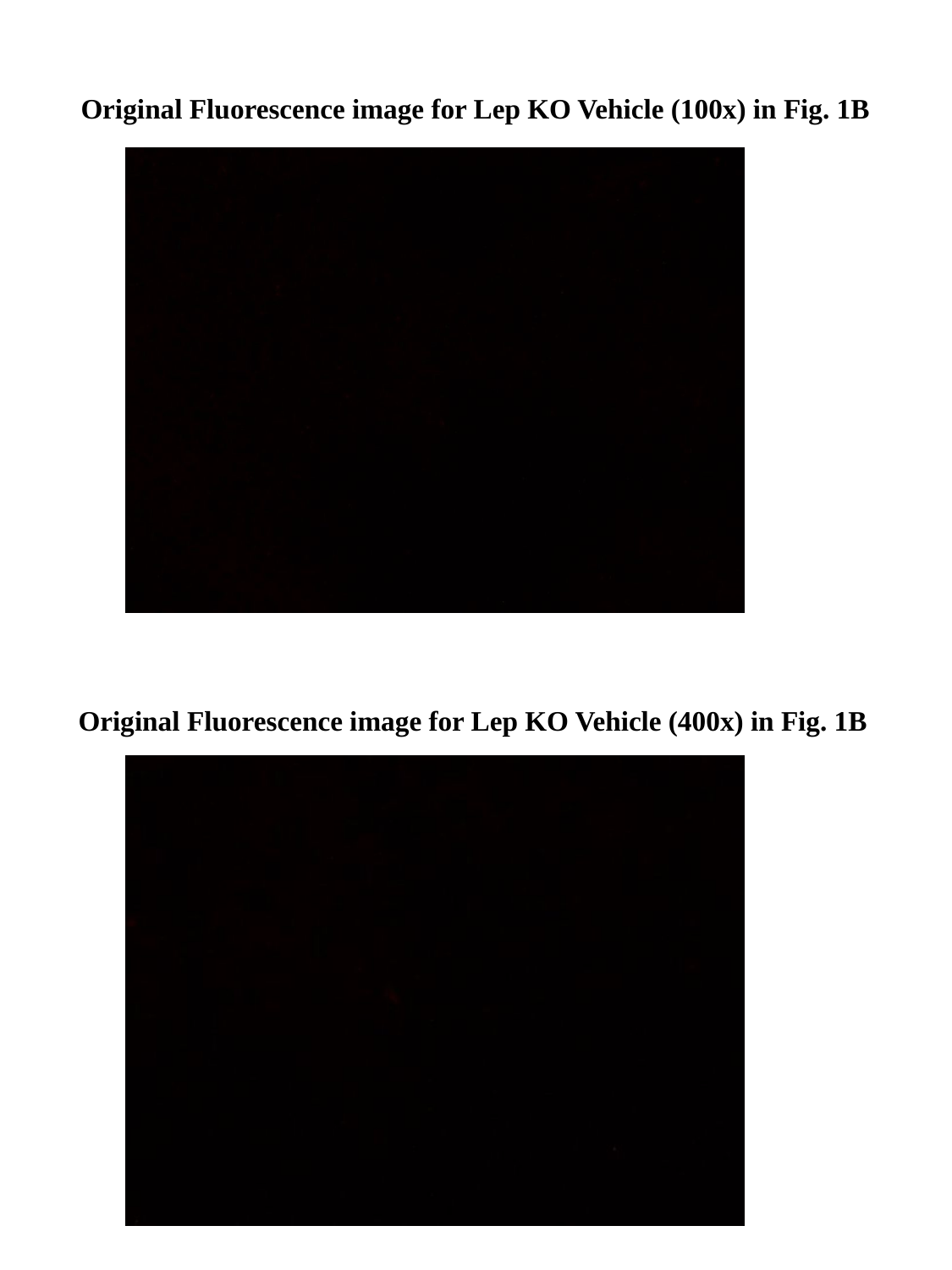

Original Fluorescence image for Lep KO Vehicle (100x) in Fig. 1B
Original Fluorescence image for Lep KO Vehicle (400x) in Fig. 1B

## Slide 8
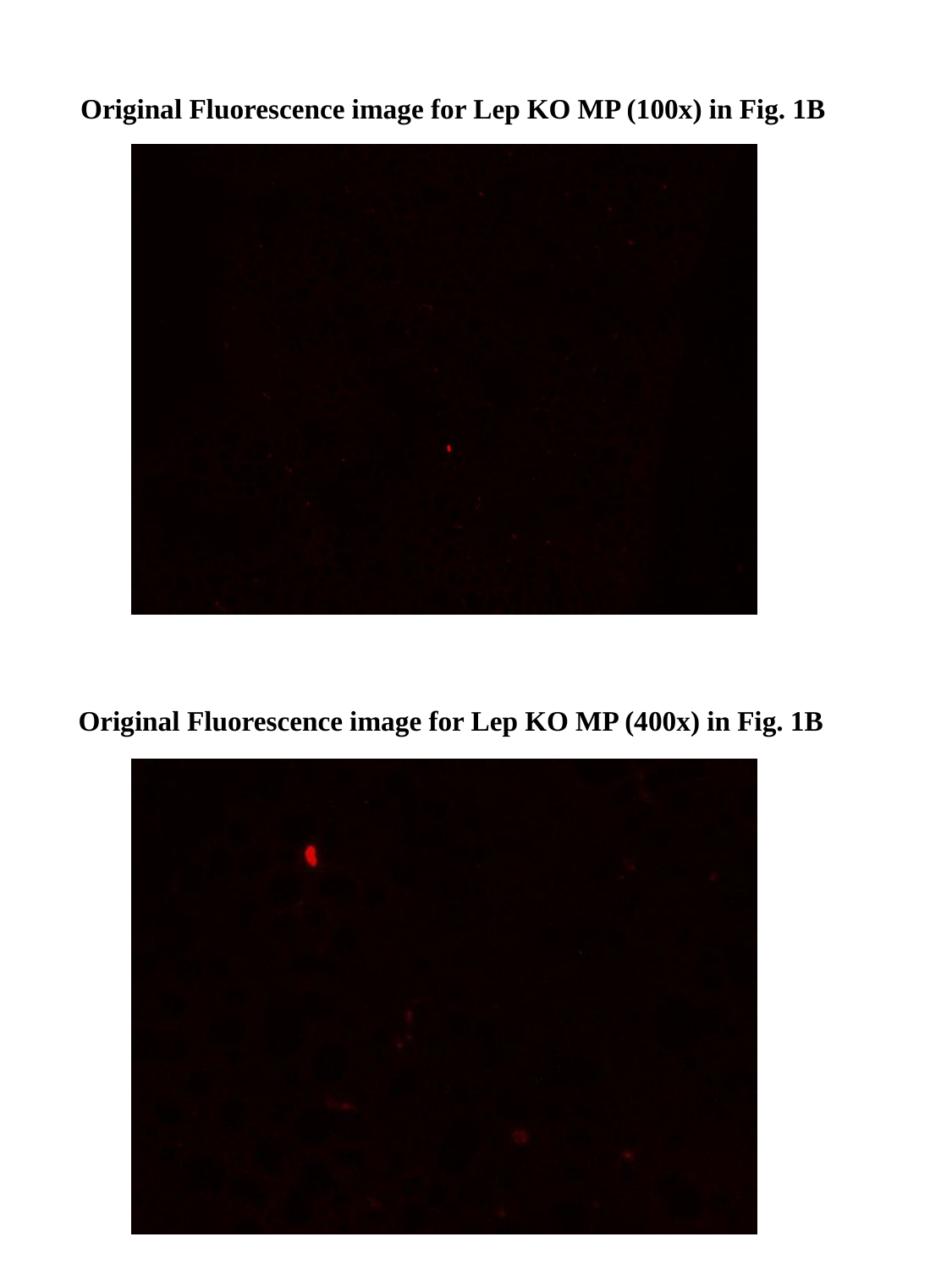

Original Fluorescence image for Lep KO MP (100x) in Fig. 1B
Original Fluorescence image for Lep KO MP (400x) in Fig. 1B

## Slide 9
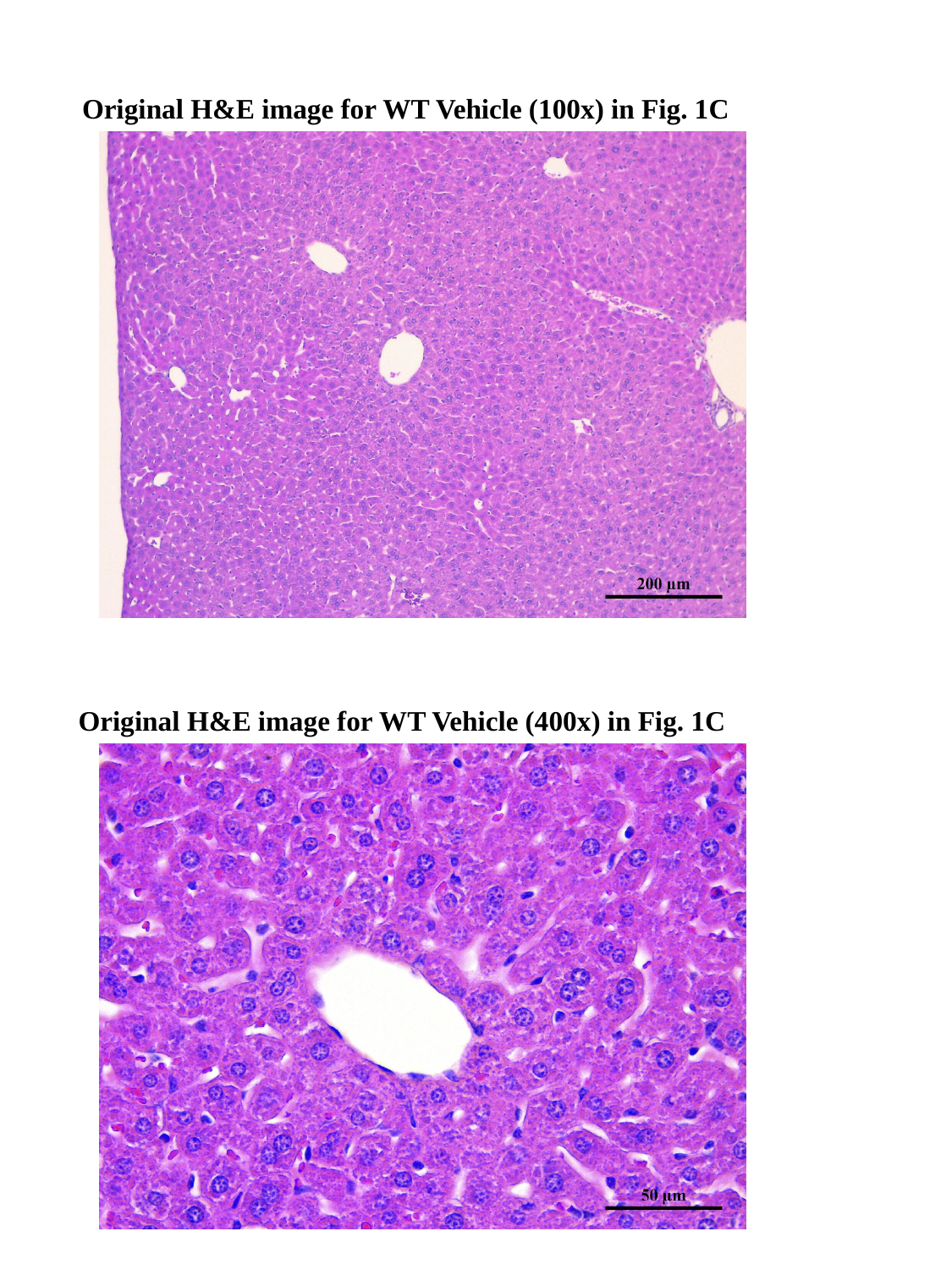

Original H&E image for WT Vehicle (100x) in Fig. 1C
Original H&E image for WT Vehicle (400x) in Fig. 1C

## Slide 10
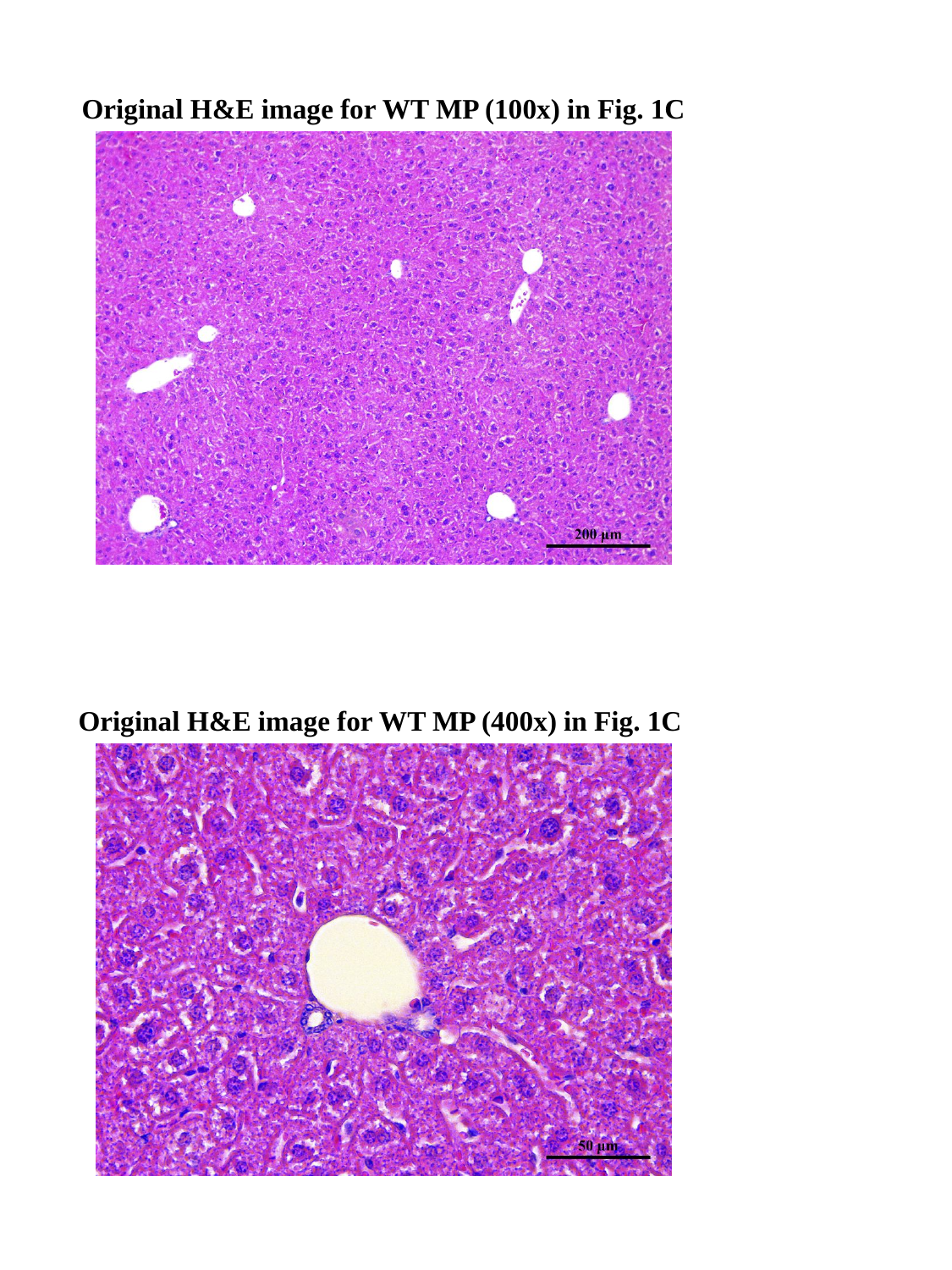

Original H&E image for WT MP (100x) in Fig. 1C
Original H&E image for WT MP (400x) in Fig. 1C

## Slide 11
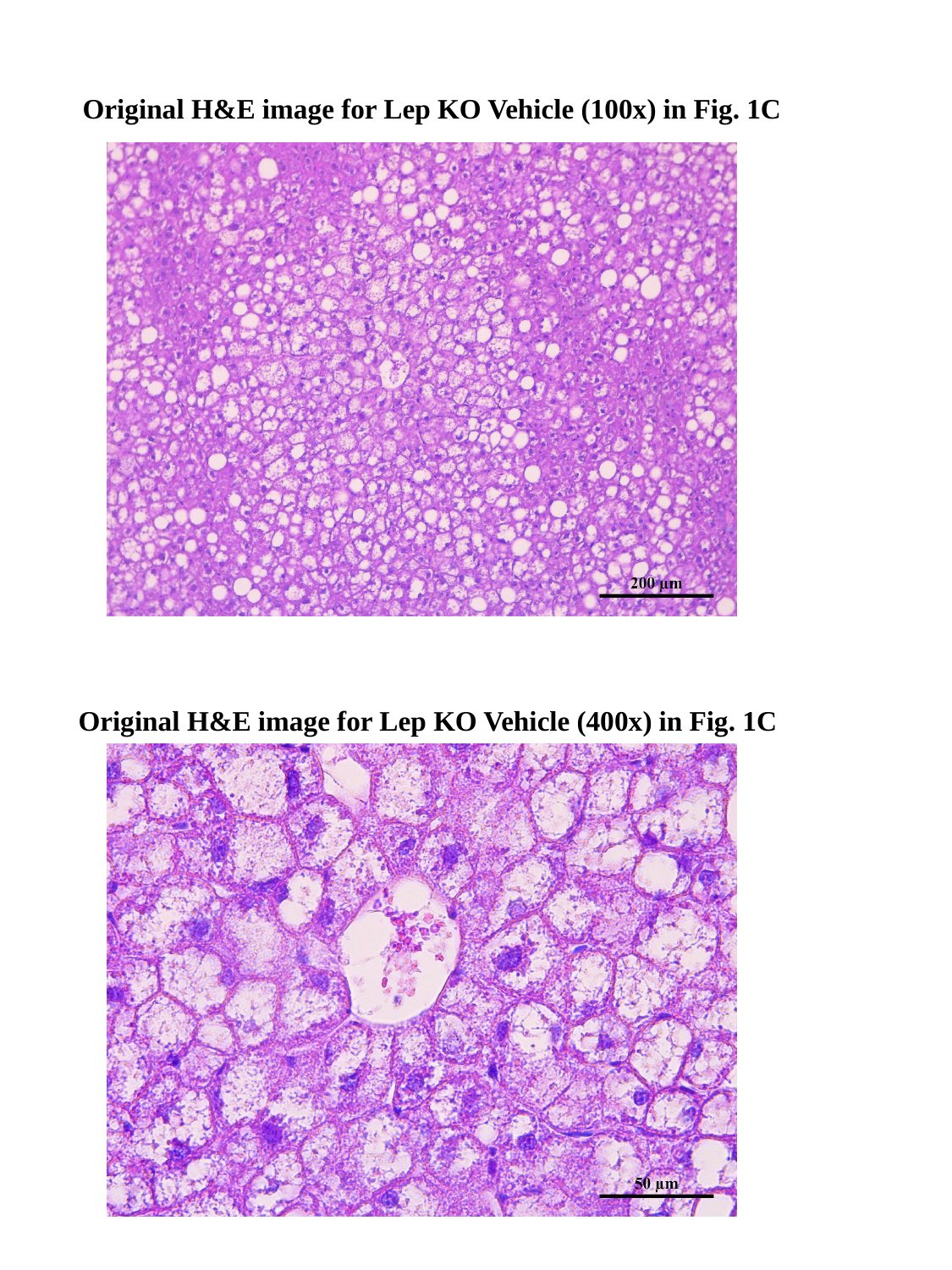

Original H&E image for Lep KO Vehicle (100x) in Fig. 1C
Original H&E image for Lep KO Vehicle (400x) in Fig. 1C

## Slide 12
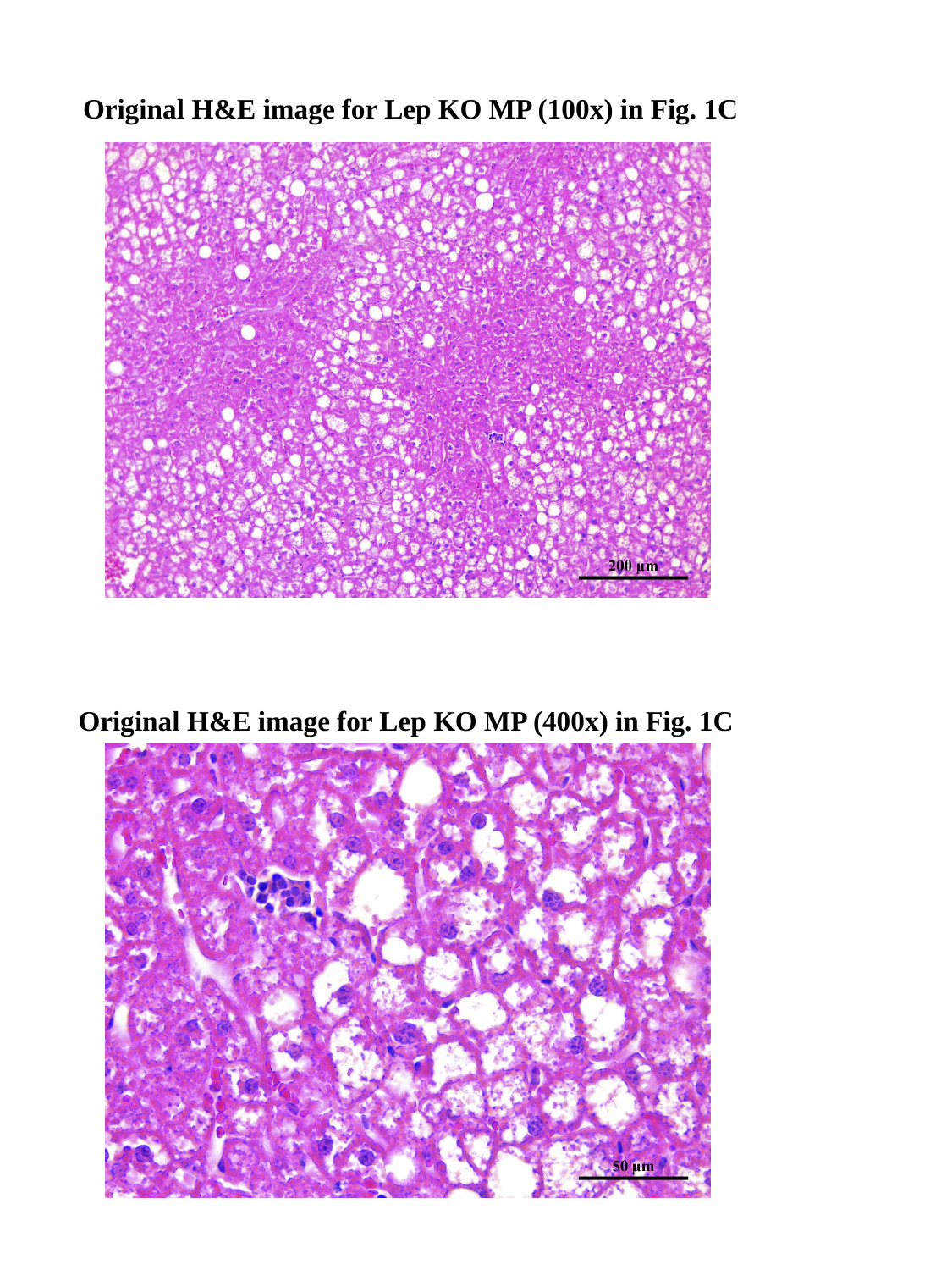

Original H&E image for Lep KO MP (100x) in Fig. 1C
Original H&E image for Lep KO MP (400x) in Fig. 1C
