# Supplementary material for: Dysbiosis of gut microbiota in C57BL/6-Lepem1hwl/Korl mice during microplastics-caused hepatic metabolism disruption
Source: PLoS One. 2025 Nov 20;20(11):e0336627. doi: 10.1371/journal.pone.0336627 (PMC12633882; doi:10.1371/journal.pone.0336627)
Supplement: Attachment — Submitted filename: Respose letter (PONE-D-25-33830)-V5.docx [file pone.0336627.s003.docx]

PUSAN NATIONAL UNIVERSITY

DEPARTMENT OF BIOMATERIALS SCIENCE

1268-50 Samrangjin-ro, Samrangjin-eup, Miryang-si, Gyeongsangnam-do 50463, Rep. of Korea

TEL: +82-55-350-5388, FAX: +82-55-350-5389, E-MAIL: dyhwang@pusan.ac.kr

September 29, 2025

To. Editorial Office

*PLoS ONE*

Sayed Haidar Abbas Raza

Manuscript No.: PONE-D-25-33830

Title: Dysbiosis of gut microbiota in C57BL/6-Lepem1hwl/Korl mice during microplastics-caused hepatic metabolism disruption

Authors:  Yu Sang Choi, Yu Jeong Roh, Ji Eun Kim, Hee Jin Song, Ayun Seol, Su Jeong Lim, Su Ha Wang, Ji Eun Sung, Ye Eun Ryu, Ye Ryeong Kim, Hyun Su Park, Hong Joo Son, Yu-Jin Kim, Eun Sang Jung, Sunho Park and Dae Youn Hwang

Dear Editor:

Now, I have revised our manuscript based on all comments raised by reviewers, and resulting revised manuscript have resubmitted for publication in the *PLoS ONE.* Enclosed is also involved a list of response against comments suggested by reviewers. Thank you very much for your cooperation.

Sincerely

Dae Youn Hwang, Professor and Director

Department of Biomaterials Science

College of Natural Resources & Life Science,

Pusan National University,

1268-50 Samnangjin-ro, Samnangjin-eup, Miryang-si,

Gyeongsangnam-do, 627-706,

Korea

[e-mail] dyhwang@pusan.ac.kr

[Te] : 82-55-350-5388

[Fax] : 82-55-350-5389

**Response to comments**

**Journal Requirements :**

*When submitting your revision, we need you to address these additional requirements.*

*1.Please ensure that your manuscript meets PLOS ONE's style requirements, including those for file naming. The PLOS ONE style templates can be found at*

<https://journals.plos.org/plosone/s/file?id=wjVg/PLOSOne_formatting_sample_main_body.pdf> and

[https://journals.plos.org/plosone/s/file?id=ba62/PLOSOne_formatting_sample_title_authors_affiliations.pdf](https://track.editorialmanager.com/CL0/https:%2F%2Fjournals.plos.org%2Fplosone%2Fs%2Ffile%3Fid=ba62%2FPLOSOne_formatting_sample_title_authors_affiliations.pdf/1/010f0198c71b1638-f7ba2a41-4cfe-4fa8-8022-d8e3e7b0cfa1-000000/sNWmVeolsAl8jYZYdq0pUjEFsMwGoV5tWeDz3_g5QFM=225)

☞ According to your comments, the manuscript has been corrected to match the PLOS ONE style.

*2. Please note that funding information should not appear in any section or other areas of your manuscript. We will only publish funding information present in the Funding Statement section of the online submission form. Please remove any funding-related text from the manuscript.*

☞ They have been deleted from manuscript.

*3. We note that the grant information you provided in the ‘Funding Information’ and ‘Financial Disclosure’ sections do not match.*

*When you resubmit, please ensure that you provide the correct grant numbers for the awards you received for your study in the ‘Funding Information’ section.*

☞ We have corrected to include the gran number.

4. *We note that your Data Availability Statement is currently as follows: All relevant data are within the manuscript and in Supporting Information files.*

*Please confirm at this time whether or not your submission contains all raw data required to replicate the results of your study. Authors must share the “minimal data set” for their submission. PLOS defines the minimal data set to consist of the data required to replicate all study findings reported in the article, as well as related metadata and methods (*[*https://journals.plos.org/plosone/s/data-availability#loc-minimal-data-set-definition*](https://journals.plos.org/plosone/s/data-availability#loc-minimal-data-set-definition)*).*

*For example, authors should submit the following data:*

*- The values behind the means, standard deviations and other measures reported;*

*- The values used to build graphs;*

*- The points extracted from images for analysis.*

*Authors do not need to submit their entire data set if only a portion of the data was used in the reported study.*

*If your submission does not contain these data, please either upload them as Supporting Information files or deposit them to a stable, public repository and provide us with the relevant URLs, DOIs, or accession numbers. For a list of recommended repositories, please see* [*https://journals.plos.org/plosone/s/recommended-repositories.*](https://journals.plos.org/plosone/s/recommended-repositories.)

*If there are ethical or legal restrictions on sharing a de-identified data set, please explain them in detail (e.g., data contain potentially sensitive information, data are owned by a third-party organization, etc.) and who has imposed them (e.g., an ethics committee). Please also provide contact information for a data access committee, ethics committee, or other institutional body to which data requests may be sent. If data are owned by a third party, please indicate how others may request data access.*

☞ According to your comments, the manuscript has been corrected to match the PLOS ONE style.

*5. Please include your full ethics statement in the ‘Methods’ section of your manuscript file. In your statement, please include the full name of the IRB or ethics committee who approved or waived your study, as well as whether or not you obtained informed written or verbal consent. If consent was waived for your study, please include this information in your statement as well.*

☞ Above issue have been already described in Material and methods section (line 81-83) as follows;

“The Pusan National University-Institutional Animal Care and Use Committee (PNU-IACUC) approved the protocols of the animal experiments based on ethical procedures for scientific care (Approval Number PNU-2022-0191 and PNU-2023-0350).”

6. *Please ensure that you refer to Figure 5 in your text as, if accepted, production will need this reference to link the reader to the figure.*

☞ This is our mistake. We have citied Figure 5 in Results section (line 218-221) as follows;

“Cluster separation was observed between the Vehicle-treated WT mice and MP-treated WT mice and between the Vehicle-treated Lep KO mice and MP-treated Lep KO mice in the unweighted and weighted UniFrac distance (Figure 5A and B).”

7. *If the reviewer comments include a recommendation to cite specific previously published works, please review and evaluate these publications to determine whether they are relevant and should be cited. There is no requirement to cite these works unless the editor has indicated otherwise.*

☞ Thank you for your advice.

**Reviewer #1 :**

*The study is well-designed, presents novel findings, and is clearly written. Methodology is sound, and results are supported by data. I recommend publication without major revisions. I am happy to endorse the manuscript.*

☞ Thank you very much.

**Reviewer #2 :**

*This study investigates whether long-term oral exposure to polystyrene microplastics (PS-MP) alters gut microbiota composition in leptin-deficient (Lep KO) mice with known hepatic metabolism disruption. WT and Lep KO mice were treated with PS-MP or vehicle for nine weeks, after which liver histology and 16S rRNA sequencing of fecal samples were performed. MP-treated Lep KO mice showed MP accumulation in liver tissue and reduced hepatic steatosis. Microbiota profiling revealed significant shifts at both the phylum and genus levels, with Lep KO mice showing marked increases in Candidatus Melainabacteria, Deferribacterota, and several genera including Lactobacillus and Ruminococcus. Alpha diversity (Chao1) decreased in both MP-treated WT and Lep KO mice, while Shannon diversity increased only in MP-treated Lep KO mice. PCoA analysis showed clear clustering differences between treatment groups. The authors conclude that MP-induced hepatic metabolism disruption is associated with dysbiosis of gut microbiota in obese Lep KO mice. The manuscript is well written but I have a few concerns*

*1. Sample size is small with only six mice per group for microbiota analysis. Fecal samples were pooled, preventing statistical assessment of individual variation and limiting the robustness of diversity and abundance comparisons.*

☞ This is good point. Actually, the number of animal per group has been considered as key factor to ensure the reliability of the data from the animal study. However, since analyzing samples obtained from a large number of animals requires a large economic cost, most papers commonly use the method of pooling fecal samples. Please understand this situation.

Meanwhile, to address above issues, we have added the justification for the total number of animals in Materials and methods section (line 102-105) as follows;

“To ensure the reliability of the data from the animal study, the total number of animals was determined as 24 using G‑POWER 3.1.9.7 (Heinrich‑Heine‑Universität Düsseldorf, Germany) with the α error probability of 0.05, effect size of 0.9 and a power of 0.95.”

In addition, the problem of data analysis due to the pooling of samples was added to the Conclusion (line 336-340) as a limitation of the study as follows;

“Also, six mice per group used for microbiota analyses in our study can be considered as another limitations to be small in sample size although fecal samples were pooled to prevent statistical evaluation of individual variants and to limit the robustness of diversity and abundance comparisons.”

*2. The study shows* *correlations between MP-induced hepatic disruption and gut microbiota changes but does not establish causality*.

☞ According to your comments, we have further described the paragraph for causality in Discussion (line 313-323) as follows;

“Generally, the scientific verification of causality for biological process requires an analysis of timativity, specificity, dose-reactivityitamins, and reversibility [50]. But, this study did not fully demonstrate causality between MP-induced hepatic disruption and gut microbiota changes although it shows the correlation between them. The results of our study were provided the evidences for the temporality and specificity between them. The temporality were demonstrated by the sequential change in the microbiota following disturbance of the liver, while specificity was proven to be a major changes in only manipulations targeting the liver pathway as described in previous studies [51,52]. However, our results do not present evidence for dose-reactivity and reversibility between them. To demonstrate the dose-reactivity, it is necessary to further analyze the dose-dependent response between MP-induced liver damages and changes of microbiota. Also, the reversibility can be demonstrated by whether recovery of liver damage is linked to recovery of microbiota composition.”

3. *Reduction in lipid droplets/steatosis in MP-treated Lep KO mice contradicts expectations and is not sufficiently explained mechanistically.*

☞ According to your comments, we have further described the paragraph for contradicts expectations in Discussion (line 244-254) as follows;

“In the MP-treated Lep KO mice of this study, the number of lipid droplets, NAFLD score and steatosis area were significantly reduced, contrary to expectations although the enhancements of them were detected in MP-treated WT mice [21]. However, the results of significant increase on these factors were detected in only ICR mice after MPs treatment with particle size of less than 50 μm, and HFD-induced obesity mouse model after treatment of an MPs [32-36]. These difference between Lep KO mice and HFD-induced obesity mice after MP treatment is thought to be related to the diversity of obesity-inducing mechanisms because some genetic and environmental factors are considered major causes of these diseases [21]. Especially, the previous study suggest that the fatty acid oxidation controlled by PPARα can be considered one of the key potential molecular mechanism involved in the impacts of MP on the hepatic lipid accumulation among four mechanism that consist of an uptake of circulating lipids, de novo lipogenesis (DNL), fatty acid oxidation (FAO), and an exportation of lipids [37,38].”

4. *16S rRNA sequencing provides taxonomic shifts, but no metagenomic or metabolomic analysis to link microbiota changes with functional metabolic* *consequences.*

☞ This is good point. As you suggestion, it is important and ideal to link microbiota changes to functional metabolic consequences. However, we could not attempt these analyses in this study because they require a lot of time and economic costs. Please understand this situation.

Therefore, these issues have been further described as limitation of our study in Conclusion (line 340-341) as follows;

“Furthermore, the metagenomic or metabolomic analyses are needed as a further study to link microbiota changes with functional metabolic consequences.”

5. *The relevance of specific taxa changes (e.g., large Lactobacillus increase) to liver metabolism or obesity is not well integrated into the discussion.*

☞ According to your comments, we have further described the paragraph for above issues in Discussion (line 308-312) as follows;

“Among microorganisms changed by MP treatment, *Firmicutes* and *Bacteroidetes* was closely associated with obesity. Obese patients showed the imbalance of microbiota with increased *Firmicutes* and decreased *Bacteroidetes*, and these changes exacerbate an obesity and metabolic diseases through stimulating inflammation, energy metabolism changes, and fat accumulation [48,49].”

6. *Housing conditions, cage effects, and diet could influence microbiota, but these are not discussed or statistically adjusted for.*

☞ This is good point. According to your comments, we have further described the paragraph for above issues in Discussion (line 232-235) as follows;

“However, it must be taken into account that changes in fecal microbiota of experimental animals may have the impact of various breeding environments including diet, drinking water, temperature, humidity, lighting cycle and microbial distribution.”

7. *Some key data (e.g., individual animal data points, variability measures for pooled samples) are missing.*

☞ According to your comments, we have uploaded the some key data as Supporting Information files based on the request of the Editorial Office.

8. *MP accumulation in liver shown qualitatively by fluorescence microscopy but not quantified to correlate with microbiota changes.*

☞ According to your comments, we have added the data of MP accumulation into Figure 1B as following;
